# The Complete Multipartite Genome Sequence of *Cupriavidus necator* JMP134, a Versatile Pollutant Degrader

**DOI:** 10.1371/journal.pone.0009729

**Published:** 2010-03-22

**Authors:** Athanasios Lykidis, Danilo Pérez-Pantoja, Thomas Ledger, Kostantinos Mavromatis, Iain J. Anderson, Natalia N. Ivanova, Sean D. Hooper, Alla Lapidus, Susan Lucas, Bernardo González, Nikos C. Kyrpides

**Affiliations:** 1 Department of Energy (DOE)-Joint Genome Institute, Walnut Creek, California, United States of America; 2 Departamento de Genética Molecular y Microbiología, Facultad de Ciencias Biológicas, NM-EMBA, NM-PFG, and CASEB, P. Universidad Católica de Chile, Santiago, Chile; 3 Facultad de Ingeniería y Ciencia, Universidad Adolfo Ibáñez, Santiago, Chile; University of Hyderabad, India

## Abstract

**Background:**

*Cupriavidus necator* JMP134 is a Gram-negative β-proteobacterium able to grow on a variety of aromatic and chloroaromatic compounds as its sole carbon and energy source.

**Methodology/Principal Findings:**

Its genome consists of four replicons (two chromosomes and two plasmids) containing a total of 6631 protein coding genes. Comparative analysis identified 1910 core genes common to the four genomes compared (*C. necator* JMP134, *C. necator* H16, *C. metallidurans* CH34, *R. solanacearum* GMI1000). Although secondary chromosomes found in the *Cupriavidus*, *Ralstonia*, and *Burkholderia* lineages are all derived from plasmids, analyses of the plasmid partition proteins located on those chromosomes indicate that different plasmids gave rise to the secondary chromosomes in each lineage. The *C. necator* JMP134 genome contains 300 genes putatively involved in the catabolism of aromatic compounds and encodes most of the central ring-cleavage pathways. This strain also shows additional metabolic capabilities towards alicyclic compounds and the potential for catabolism of almost all proteinogenic amino acids. This remarkable catabolic potential seems to be sustained by a high degree of genetic redundancy, most probably enabling this catabolically versatile bacterium with different levels of metabolic responses and alternative regulation necessary to cope with a challenging environment. From the comparison of *Cupriavidus* genomes, it is possible to state that a broad metabolic capability is a general trait for *Cupriavidus* genus, however certain specialization towards a nutritional niche (xenobiotics degradation, chemolithoautotrophy or symbiotic nitrogen fixation) seems to be shaped mostly by the acquisition of “specialized” plasmids.

**Conclusions/Significance:**

The availability of the complete genome sequence for *C. necator* JMP134 provides the groundwork for further elucidation of the mechanisms and regulation of chloroaromatic compound biodegradation.

## Introduction

*Cupriavidus necator* JMP134 (formerly *Ralstonia eutropha* JMP134) is a Gram-negative β-proteobacterium able to degrade a variety of chloroaromatic compounds and chemically-related pollutants. It was originally isolated based on its ability to use 2,4 dichlorophenoxyacetic acid (2,4-D) as a sole carbon and energy source [1]. In addition to 2,4-D, this strain can also grow on a variety of aromatic substrates, such as 4-chloro-2-methylphenoxyacetate (MCPA), 3-chlorobenzoic acid (3-CB) [2], 2,4,6-trichlorophenol [3], and 4-fluorobenzoate [4]. The genes necessary for 2,4-D utilization have been identified. They are located in two clusters on plasmid pPJ4: *tfd*_I_ and *tfd*_II_ [5], [6], [7], [8]. The sequence and analysis of plasmid pJP4 was reported and a congruent model for bacterial adaptation to chloroaromatic pollutants was proposed [9]. According to this model, catabolic gene clusters assemble in a modular manner into broad-host-range plasmid backbones by means of repeated chromosomal capture events.

*Cupriavidus* and related *Burkholderia* genomes are typically multipartite, composed of two large replicons (chromosomes) accompanied by classical plasmids. Previous work with *Burkholderia xenovorans* LB400 revealed a differential gene distribution with core functions preferentially encoded by the larger chromosome and secondary functions by the smaller [10]. It has been proposed that the secondary chromosomes in many bacteria originated from ancestral plasmids which, in turn, had been the recipient of genes transferred earlier from ancestral primary chromosomes [11]. The existence of multiple *Cupriavidus* and *Burkholderia* genomes provides the opportunity for comparative studies that will lead to a better understanding of the evolutionary mechanisms for the formation of multipartite genomes and the relation with biodegradation abilities.

## Materials and Methods

### Genome sequencing and assembly

The complete genome of *C. necator* JMP134 was sequenced at the Joint Genome Institute using a combination of 3 kb and fosmid (40 kb) libraries. Library construction, sequencing, finishing, and automated annotation steps were performed as described at the JGI web page (http://www.jgi.doe.gov/sequencing/index.html). Gene prediction was performed using CRITICA [12] and Glimmer [13] followed by manual inspection of the automatically predicted gene models. Predicted coding sequences (CDSs) were manually analyzed and evaluated using an Integrated Microbial Genomes (IMG) annotation pipeline (http://img.jgi.doe.gov) [14]. CLUSTALW was used for sequence alignments [15]; phylogenetic trees were built using Phylip.

### Genome analysis

Functional annotation and comparative analysis of *C. necator* with related organisms was performed using a set of tools available in IMG. Unique and orthologous *C. necator* genes were identified using BLASTp (reciprocal best BLASTp hits with cutoff scores of *E* <10^−5^ and 60% identity). Signal peptide cleavage sites were identified using SignalP 3.0 [16] and transmembrane proteins were predicted using TMHMM [17], both with their default settings. Synteny plots were made using Promer, a subroutine of Mummer [13].

### GenBank accession numbers

The sequences of the four genomic replicons described here have been deposited in GenBank (accession numbers CP000090-CP000093), and the project information to the GenomesOnline Database (Gc00292) [18].

## Results and Discussion

### General genome features

**Figure 1:**
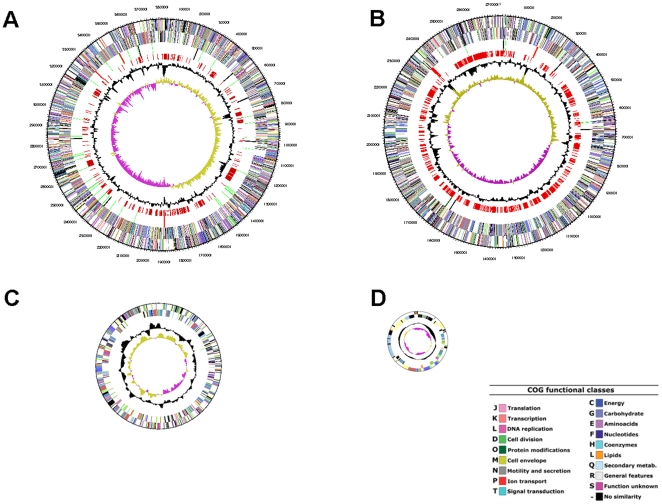
Circular representations of the four replicons of the *C. necator* genome. 1A: chromosome 1; 1B: chromosome 2; 1C: megaplasmid; 1D: plasmid pPJ4. Circle 1 (from outside to inside): COG assignments for CDSs on the plus strand. Circle 2: COG assignments for CDSs on the minus strand. Circle 3: RNA genes (green  =  tRNAs; red  =  rRNAs; black  =  other RNAs). Circle 4 (for chromosome 1 and 2, only): genes not found in *C. eutropha* H16, *C. metallidurans* CH34, or *R. solanacearum* GMI1000. Circle 5: % G+C. Circle 6: GC skew (G-C/G+C). Colors indicate the following: dark gray, hypothetical proteins; light gray, conserved hypothetical and unknown function; brown, general function prediction; red, replication and repair; green, energy metabolism; blue, carbon and carbohydrate metabolism; cyan, lipid metabolism; magenta, transcription; yellow, translation; orange, amino acid metabolism; pink, metabolism of cofactors and vitamins; light red, purine and pyrimidine metabolism; lavender, signal transduction; and blue sky, cellular processes.

The genome of *C. necator* JMP134 consists of four DNA molecules: two circular chromosomes and two plasmids (Table 1 and Figure 1). The four replicons combined contain 6,631 protein coding sequences (CDSs), of which 4,898 (73.8%) could be assigned a putative function. There are 87 RNA genes including 66 tRNAs and six rRNA loci, each arranged in the order of 5S-23S-16S. Also identified were 83 pseudogenes. Analysis of the distribution of genes representing major functional categories reveals that chromosome 1 encodes most of the key functions required for transcription, translation, and DNA replication, while chromosome 2 encodes functions involved in energy production and conversion, secondary metabolism, and amino acid transport and metabolism.

**Table 1:** Comparative genome statistics of five β-proteobacteria.

	Chromosome 1				Chromosome 2				Plasmid 1				Plasmid 2			
	Length (Mb)	CDSs	16S RNAs	tRNAs	Length (Mb)	CDSs	16S RNAs	tRNAs	Length (Mb)	CDSs	16S RNAs	tRNAs	Length (Mb)	CDSs	16S RNAs	tRNAs
*C. necator* JMP134	3.80	3537	3	54	2.72	2449	3	11	0.63	555	-	1	0.08	90	-	-
*C. eutropha* H16	4.05	3723	3	49	2.91	2571	2	5	0.45	424	-	3	-	-	-	-
*C. metallidurans* CH34	3.92	3684	2	54	2.58	2341	2	8	0.23	241	-	-	0.02	164	-	-
*R. solanacearum* GMI1000	3.71	3521	3	53	2.09	1686	1	-	-	-	-	-	-	-	-	
*B. xenovorans* LB400	4.89	4615	3	57	3.36	3054	3	8	1.47	1390	-	-	-	-	-	-

### Comparative genomics

**Figure 2:**
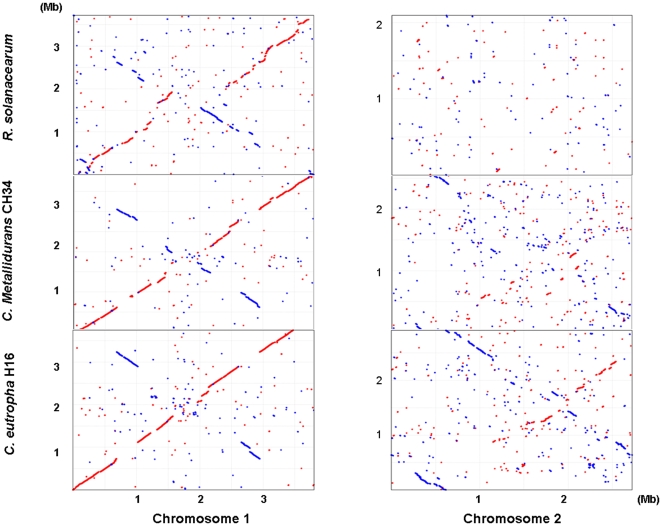
Synteny plots between *C. necator* JMP134 (horizontal axis) and *C. eutropha* H16, *C. metallidurans* CH34, and *R. solanacearum* GMI1000. Red  =  leading strand; blue  =  lagging strand.

Various comparisons were made between the genome of *C. necator* JMP134 and four other closely-related β-proteobacteria that also possess multipartite genomes (Table 1). Synteny plots comparing *C. necator* JMP134 with other closely related *Cupriavidus/Ralstonia* genomes (*C. necator* H16; *C. metallidurans* CH34; and *Ralstonia solanacearum* GM1000) reveal extensive conservation of chromosome 1 but a lack of synteny in chromosome 2 (Figure 2). The origin and evolutionary history of chromosome 2 probably includes multiple occurrences of gene duplication and lateral gene transfer (see below). Notably, in all four species chromosome 2 contains three copies of the rRNA locus, thus indicating past recombination between chromosomes 1 and 2.

**Figure 3:**
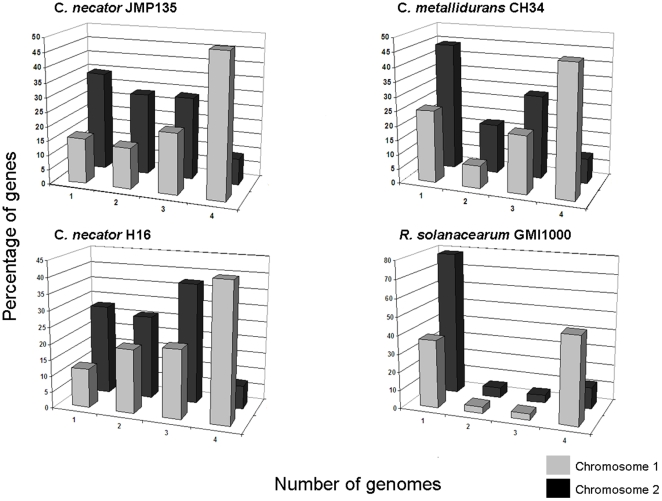
Distribution of shared and unique chromosomal genes in the genomes of three *Cupriavidus* and one *Ralstonia*. X-axis: the number of genomes (1–4) where the gene is found. Y-axis: the percentage of genes in the genome that are found in 1, 2, 3, or all 4 of the compared genomes.

These four genomes were also compared by determining the numbers of genes encoded by each that are unique to one organism and the number that are shared by two, three, or all four strains (Figure 3). Protein identity was defined conservatively using reciprocal best BLASTp hits with a cutoff of 60% identity of the amino acid sequence. By that criterion, 1910 genes are found in all four strains (1713 on chromosome 1, 197 on chromosome 2).

**Figure 4:**
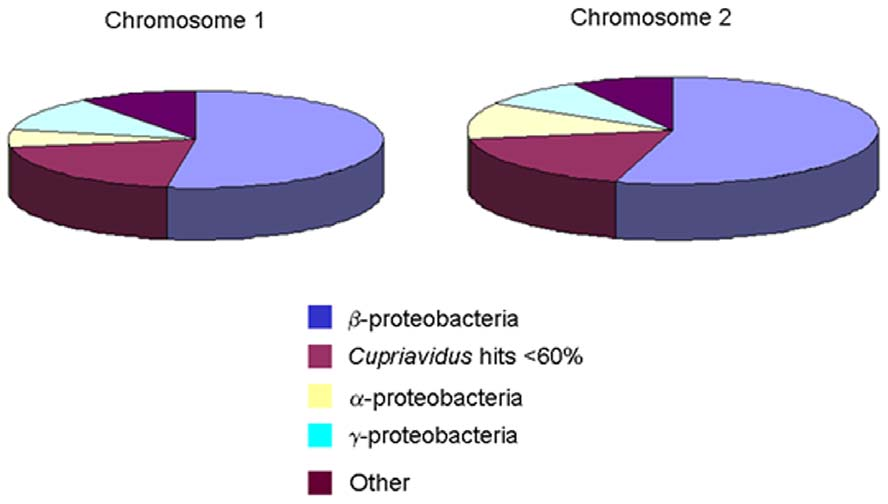
Phylogenetic distribution of the best BLASTp hits to the unique genes in *C. necator* JMP134. Unique genes are those not present in the three other strains compared.

Approximately 28.7% of the CDSs in the genome of *C. necator* JMP134 (1904 out of 6,631) were not found in any of the other three genomes. These 1904 unique genes are distributed among all four replicons: 552 on chromosome 1, 841 on chromosome 2, 432 in the megaplasmid, and 80 in plasmid pPJ4. Of the 552 unique genes on chromosome 1: 43 (8%) have no orthologs or paralogs in the current version of IMG; 87 (15%) have a best BLASTp hit within *C. necator* JMP134 indicating that they arose from gene duplication; 422 (76%) have a best BLASTp hit to other organisms within the database (Figure 4). The majority of those organisms are other β-proteobacteria, particularly *Burkholderiaceae*, with a minor percentage also from the *Alcaligenaceae* and the *Comamonadaceae* β-proteobacterial families. A sizable minority of them (∼30%) are found in other phylogenetically diverse soil bacteria. Of the 841 unique genes on chromosome 2 of *C. necator*, 47 (6%) have no orthologs or paralogs, 181 (22%) have a best BLASTp hit within the *C. necator* JMP134 genome, and 612 (73%) have a best BLASTp hit to other genomes (Figure 4). These data indicate that the evolution of these two chromosomes has involved substantial gene duplication and extensive lateral gene transfer events (preferentially with related organisms, i.e., β-proteobacteria).

To analyze the functional content of these unique genes we examined their distribution towards particular COGs (Figure S1). Excluding COGs R and S (categorized as General features and Hypothetical Functions, respectively), the data indicate that the majority of the unique genes belong to COG K. COG K refers to transcription and the majority of these unique genes are transcriptional regulators. Although the distribution of unique genes to various COG categories differs among the four organisms, a significant number of unique genes belong to signal transduction pathways (COG T, mainly histidine kinases and response regulators), energy production and conversion (COG C, mainly dehydrogenases, oxidases and hydroxylases), amino acid transport and metabolism (COG E, mainly transporters), and lipid metabolism (COG I, mainly acyl-CoA synthetases and dehydrogenases, enoyl-CoA hydratases).

Similarly, *C. eutropha* H16 has 2000 genes that are not present in any of the other three strains: 784 on chromosome 1, 956 on chromosome 2, and 258 in its megaplasmid pHG1. Interestingly, orthologs for 122 genes found in megaplasmid pHG1 are present on the chromosomes of the other two *Cupriavidus* strains: 35 in *C. necator* JMP134 and 82 in *C. metallidurans* CH34.

Of the 2,449 genes identified on chromosome 2 of *C. necator* JMP134, 460 (18.8%) have orthologs on chromosome 1 of either *C. eutropha* H16, *C. metallidurans* CH34, or *R. solanacearum*, but only 45 of them have orthologs in more than one genome.

**Figure 5:**
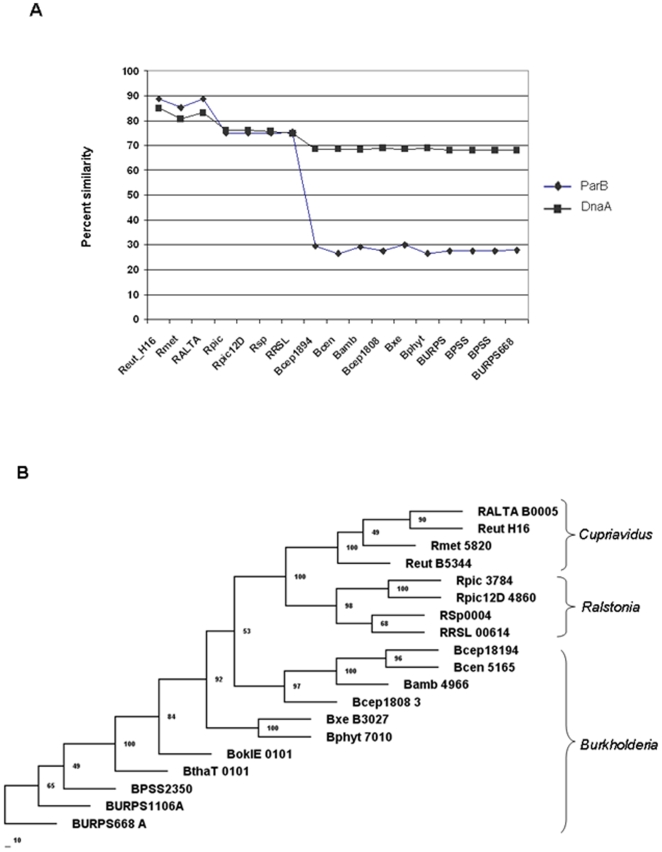
ParB protein similarity and phylogeny. A. Percent identity plots of *C. necator* ParB (Reut_B5344) and DnaA (Reut_A0001) proteins. B. ParB phylogeny. Neighbor joining tree of 19 ParB proteins. Sequences are from the following species: RALTA, *Cupriavidus taiwanensis*; Reut_H16, *Cupriavidus necator* H16; Rmet, *Cupriavidus metallidurans* CH34; Rpic, *Ralstonia pickettii* 12J; Rpic12D *Ralstonia pickettii* 12D; RSp, *Ralstonia solanacearum* GMI1000; RRSL, *Ralstonia solanacearum* UW551; Bcep18194, *Burkholderia sp*. 383; Bcen, *Burkholderia cenocepacia* AU 1054; Bamb, *Burkholderia cepacia* AMMD; Bcep1808, *Burkholderia vietnamiensis* G4; Bxe, *Burkholderia xenovorans* LB400; Bphyt, *Burkholderia phytofirmans* PsJN; BokIE, *Burkholderia oklahomensis* EO147; BthaT, *Burkholderia thailandensis* TXDOH; BPSS, *Burkholderia pseudomallei* K96243; BURPS1106, *Burkholderia pseudomallei* 1106a; BURPS668, *Burkholderia pseudomallei* 668. The corresponding tree built for ParA was similar (not shown).

The prevailing hypothesis for the origin of the secondary chromosome in the multipartite genomes of *Cupriavidus* and *Burkholderia* posits that it evolved from ancestral plasmids. We sought to determine whether these putative ancestral plasmids were the same in the *Cupriavidus*/*Ralstonia*, and *Burkholderia* lineages. Since chromosome 2 encodes homologs of ParA and ParB (proteins involved in the active partitioning of low-copy-number plasmids), we investigated the similarity and phylogenetic relationships of the ParA and ParB proteins encoded by chromosome 2 in 19 β-proteobacteria from those three genera (Figure 5). Figure 5A shows the similarity of the *C. necator* ParB and DnaA (present in chromosome 1) to the corresponding proteins of the other lineages. Although the identity of the DnaA proteins is preserved to around 70%, the identity of the ParB proteins is significantly lower among *Cupriavidus*/*Ralstonia* and *Burkholderia* species (∼28%). Phylogenetic analysis (Figure 5B) also indicates that ParB proteins from the *Cupriavidus* and *Ralstonia* lineages form distinct groups. Taken together, these data suggest that two distinct plasmids (one for *Cupriavidus*/*Ralstonia* and one for *Burkholderia*) may have been the origin of the secondary chromosomes present in the genera *Cupriavidus*/*Ralstonia*, and *Burkholderia*.

### Catabolism of aromatic compounds

We have reconstructed the metabolic pathways for aromatic compound degradation in *C. necator* JMP134, comparing the catabolic abilities found *in silico* with the range of compounds that support growth of this strain [19]. *C. necator* is able to use 60 aromatic compounds as a sole carbon and energy source. Aromatic degradation pathways have been classified to central and peripheral. Peripheral pathways transform a large variety of aromatic compounds into a few key intermediates (such as gentisate, catechol, benzoyl-CoA etc) which are subsequently degraded via the central pathways. All of the central ring-cleavage pathways for aromatic compounds known in Proteobacteria, with the exception of the homoprotocatechuate pathway, are found in this strain: the β-ketoadipate pathway, with its catechol, chlorocatechol and protocatechuate *ortho* ring-cleavage branches (*cat*, *tfd* and *pca* genes, respectively); the 4-methylcatechol *ortho* ring-cleavage pathway (*mml* genes); the gentisate ring-cleavage pathway (*mhb* genes); the phenylacetyl-CoA ring-cleavage pathway (*paa* genes); the homogentisate ring-cleavage pathway (*hmg* genes); the 2,3-dihydroxyphenylpropionate *meta* ring-cleavage pathway (*mhp* genes); the catechol *meta* ring-cleavage pathway (*phl* genes); the chlorohydroxyquinol *ortho* ring-cleavage pathway (*tcp* genes); the aminohydroquinone ring-cleavage pathway (*mnp* genes); and the 2-aminobenzoyl-CoA ring-cleavage pathway (*abm* genes).

The approximately 300 genes predicted to be directly involved in catabolism of aromatic compounds were found to be more or less equally distributed between chromosomes 1 and 2. Gene redundancy is predicted to play a significant role in the catabolic potential of *C. necator*. Redundant functions were observed in the catechol, protocatechuate, salicylate, and phenylacetyl-CoA pathways; in the degradative pathways for benzoate and chloroaromatic compounds; in some of the *p*-hydroxybenzoate and (methyl)phenols peripheral reactions; in the presence of several *meta* ring-cleavage enzymes; in other oxygenases, maleylacetate reductases and regulatory proteins. In total, the genome of *C. necator* encodes more than 70 oxygenases belonging to the main oxygenase groups that function in the catabolism of aromatic compounds. Is this extensive catabolic versatility shared by other soil bacteria? Genome-wide studies performed on *P. putida* KT2440 [20], *B. xenovorans* LB400 [10], *Rhodococcus* jostii RHA1 [21], and “*A. aromaticum*” sp. EbN1 [22] show a significant degree of catabolic versatility, based on the high number of aromatic pathways encoded, suggesting that bacteria with such capabilities may be more common in nature than previously supposed.

### Transport of aromatic compounds

A search for transporter genes in the vicinity of genes encoding aromatic degradative enzymes located ABC transporters from several families, including the family 4 ABC transporters. This group, originally identified as branched-chain amino acid transporters, has more recently been found to also transport other amino acids and urea (http://www.tcdb.org). One member of this family is known to function in transport of aromatic compounds [23]. *C. necator* JMP134 contains several family 4 ABC transporters that are predicted to transport aromatic compounds, most—but not all—of which are shared with other *Cupriavidus* strains.

One family 4 transporter (Reut_A1329-1333) shared by the three *Cupriavidus* strains is adjacent to genes involved in benzoate degradation. This one is similar to that found in the *box* operon in *Azoarcus evansii* [24], and also to an *hba* operon in *R. palustris* GCA009 that encodes hydroxybenzoate degradation [25]. Another family 4 ABC transporter (Reut_B3779-3783) adjacent to a ring-hydroxylating dioxygenase is found only in *C. necator* JMP134 and *C. eutropha* H16. In a family 4 ABC transporter found also in *C. metallidurans* CH34 and *R. solanacearum* GMI1000, the binding protein (Reut_B4017) is separated by several genes from the permease and ATPase components (Reut_B4007-4010) which are, in turn, adjacent to a gene encoding a 4-hydroxybenzoate 3-monooxygenase. However, the transporters (Reut_B3779-3783, and (Reut_B4007-4010, Reut_B4017) do not cluster with sequences related to the degradation of aromatic compounds.

Two putative aromatic compound ABC transporters that are unique to *C. necator* JMP134 are located on plasmids. One (Reut_C6326-6330) is found on the megaplasmid where it is one gene away from a putative 3-chlorobenzoate 3,4-ring-hydroxylating dioxygenase. The other (Reut_D6487-6490) is on plasmid pPJ4 [9]. However, this transporter has a high similarity to a probable urea transporter in the *C. necator* JMP134 genome (Reut_A0986- 0990) that is adjacent to urease encoding genes.

Some ABC transporter families that have not been previously known to transport aromatic compounds are found in the vicinity of aromatic degradative enzymes, including two from families 15/16 (COG0715). One full transporter (Reut_B5799-5801) and one binding protein (Reut_C6311) may be involved in aromatic compound transport. A family 2 ABC transporter (Reut_B4133-4136) may also function in aromatic compound transport as it is directly adjacent to a dioxygenase putatively involved in ring hydroxylation. The only closely related transporter found is in *Bradyrhizobium japonicum* where it, also, is adjacent to genes of aromatic catabolism.

*C. necator* JMP134 has only two members of the benzoate: proton symporter family (TC 2.A.46): Reut_A2362 that is shared with *C. metallidurans* CH34 and *R. solanacearum* GMI1000, and Reut_B5351 that is unique to strain JMP134. Also found in *C. necator* JMP134 are 13 members of a family of aromatic acid transporters—family 15 of the major facilitator superfamily (MFS). In addition, *C. necator* JMP134 has one MFS family 27 transporter and one family 30 transporter, both likely to be involved in aromatic compound uptake.

We investigated the possible presence of permease-type aromatic transporters by searching for homologs to the following proteins: BenK from *Acinetobacter baylyi* ADP-1 (the only benzoate transporter with a biochemically confirmed function); VanK, MucK, and PcaK from *A. baylyi* ADP-1 (transporters with other biochemically confirmed transport functions); and four putative transporter proteins (BenK from *Pseudomonas putida* PRS2000, PcaK from *Azoarcus* sp. EbN1, BenK from *Rhodococcus* sp. RHA1, and a putative transporter from *A*. *baylyi* ADP-1. This search identified 30 possible transporters with varying degrees of similarity to described aromatic acid transporters of this type.

### Additional metabolic features

In addition to the broad catabolic potential towards aromatic compounds, strain JMP134 degrades various other pollutants such as cyclohexanecarboxylate, tetrahydrofurfuryl alcohol and acetone. The pathways utilized for the degradation of the above compounds correspond to the ones described in other bacteria (Table S1) [26], [27], [28], [29], [30], [31], [32].

Some interesting groups of enzymes without specific physiological role are also encoded in the genome of this bacterium: (i) Bacterial dehalogenases are important in the metabolism of diverse halogenated compounds originated from natural and anthropogenic sources [33], [34], and some representatives of different kinds of dehalogenases seem to be encoded in the genome of strain JMP134. They include homologs of the hydrolytic (S)-2-haloacid dehalogenase (Reut_A1952 and Reut_B5662) and a reductive dehalogenase belonging to glutathione S-transferase (GST) superfamily (Reut_C5979), probably involved in dechlorination of 2-chloro-5-nitrophenol [19]. Additionally, two contiguous genes (Reut_A1486 and Reut_A1487) both belonging to the GST family, show high identity with ORF3 and ORF4 of the *tft* cluster involved in metabolism of 2,4,5-trichlorophenoxyacetate by *Burkholderia cepacia* AC1100 [35], suggesting a probably role as dechlorinating enzymes in catabolism of chloroaromatic compounds. (ii) Bacterial nitroreductases are flavoenzymes that catalyze the NAD(P)H-dependent reduction of the nitro groups on nitroaromatic and nitroheterocyclic compounds. These enzymes have raised a great interest due to their potential applications in bioremediation and biocatalysis [36]. At least four nitroreductases probably involved in metabolism of nitroaromatic or nitroheterocyclic compounds are encoded in the genome of strain JMP134: Reut_B3607, Reut_C6301, Reut_C5940 and Reut_C5984. The last three of them are encoded by genes located in the megaplasmid and without close homologs in the rest of *Cupriavidus*/*Ralstonia* strains, suggesting that this replicon could be specialized in catabolism of nitroaromatic compounds, besides 3-nitrophenol catabolism [19]. (iii) Baeyer-Villiger monooxygenases (BVMO) are a type of flavoproteins that play a role in hydroxylation of either alicyclic, aliphatic, or aryl ketones to form a corresponding ester, which can easily be hydrolyzed. These enzymes attract a huge interest on industrial applications since they are able to perform highly regio- and enantio- selective oxygenations on several substrates. The strain JMP134 has four genes putatively encoding BVMO (Reut_B5461, Reut_C6279, Reut_B4935 and Reut_B5155) that are scattered across the genome and are present in clusters with other genes coding for subsequent metabolism downstream of the monooxygenase reaction (i.e., esterases, hydrolases and alcohol/aldehyde dehydrogenases) but this fact does not shed enough light about their physiological substrates. A few related homologs are also found in the rest of *Cupriavidus* genomes.

### Degradation of amino acids

*C. necator* JMP134 is able to grow on all the proteinogenic amino acids except glycine, methionine, arginine and lysine [37]. This pattern of amino acids utilization is identical for *C. necator* H16 and slightly different for *C. metallidurans* CH34, which is unable to use tryptophane and cysteine but grows on glycine and lysine [37]. It should be noted that glutamine and asparagine were not included in this study [37].

The inability of strain JMP134 to grow on arginine is consistent with the absence of genes coding for any of the four arginine catabolic pathways described in bacteria: the arginine deiminase, the arginine decarboxylase, the arginine dehydrogenase and the arginine succinyltransferase pathway [38]. These genes are also absent in *Cupriavidus*/*Ralstonia* strains H16, CH34, LMG19424, GMI1000 and 12J. On the other hand, the absence of genes coding for the cadaverine pathway, the aminovalerate pathway and the aminoadipate pathway involved in degradation of lysine [39] is consistent with the inability of this bacterium to grow on this amino acid. Similarly, these genes are not found in the rest of *Cupriavidus*/*Ralstonia* strains, but the presence of a putative ornithine/lysine/arginine decarboxylase (Reut_A0689, H16_A2930, Rmet_2754, RALTA_A2412, RSc2365, Rpic_2578) in all the *Cupriavidus*/*Ralstonia* strains is intriguing, since the ability to grow on these amino acids is not a metabolic trait of these genera. An explanation for this apparent inconsistency is that the role of this putative ornithine/lysine/arginine decarboxylase in *Cupriavidus*/*Ralstonia* strains is exclusively in acid resistance and not in catabolism since this kind of amino acids decarboxylases are acid-induced and are part of an enzymatic system in *E. coli* that contributes to making this organism acid-resistant [40].

The inability of use methionine as growth substrate by JMP134 and the rest of *Cupriavidus*/*Ralstonia* strains is consistent with the absence of L-methionine γ-lyase, a pyridoxal 5′-phosphate-dependent enzyme that catalyzes the direct conversion of L-methionine into α-ketobutyrate, methanethiol, and ammonia [41].

The presence of a putative glycine cleavage enzyme system in *C. necator* JMP134, encoded by the *gcvTHP* genes (Table S1), catalyzing the oxidative cleavage of glycine to CO_2_, NH_3_ and transferring a one-carbon unit to tetrahydrofolate would be contradictory with the inability of this strain to grow in glycine. However, it should be noted that the metabolism of one-carbon compounds in *C. necator* JMP134 is not enough to support growth on these compounds as sole carbon source and they are only used as an auxiliary energy source [37], in contrast with chemolithoautotroph strains as H16 and CH34 (See energy metabolism section).

Glutamine is also included among the amino acids that are not supporting growth of *C. necator* JMP134, since a glutaminase encoding-gene, enabling the transformation of glutamine to glutamate, is not found in this strain, although is present in strains CH34 and GMI1000. A gene encoding a bifunctional proline dehydrogenase/pyrroline-5-carboxylate dehydrogenase, catalyzing the four-electron oxidation of proline to glutamate, is found in the genome of strain JMP134 (Table S1) and the rest of *Cupriavidus*/*Ralstonia* strains allowing the utilization of proline by these bacteria. According to this trait, a glutamate dehydrogenase-encoding gene, converting glutamate to α-ketoglutarate and thus directly feeding the tricarboxylic acids cycle is found in strain JMP134 (Table S1) and the rest of *Cupriavidus* strains, but not in strains 12J and GMI1000.

The presence in strain JMP134 of an L-asparaginase-encoding gene, enabling the hydrolysis of L-asparagine to L-aspartate and ammonia (Table S1), would suggest that this strain is able to use this amino acid as sole carbon and energy source. This gene is also encoded in the genomes of the rest of *Cupriavidus* strains but not in strains 12J and GMI1000. The formed aspartate can be metabolized through conversion to oxaloacetate by L-aspartate oxidase (NadB), or to fumarate by aspartate-ammonia-lyase (AspA) (Table 1). The presence of an L-aspartate oxidase-encoding gene is common to the rest of *Cupriavidus*/*Ralstonia* strains, but the aspartate-ammonia-lyase is a peculiarity of *C. necator* JMP134. Alternatively, aspartate may be transformed to alanine by an aspartate 1-decarboxylase, however a gene encoding this enzyme was not found in *C. necator* JMP134, in contrast with strains H16, LMG19424, 12J and GMI1000 that harbor an aspartate 1-decarboxylase-encoding gene.

The genomic analysis of strain JMP134 suggests that L-alanine can be degraded by two different pathways. L-alanine can be directly degraded to pyruvate and ammonia by a NADH-dependent L-alanine dehydrogenase or converted to D-alanine by an alanine racemase and subsequently degraded to pyruvate and ammonia via D-alanine dehydrogenase (Table S1) [42]. The D-alanine pathway seems to be shared by the rest of *Cupriavidus*/*Ralstonia* strains, but the L-alanine dehydrogenase is only found in strains H16 and JMP134.

Serine and threonine seem to be used as carbon source by strain JMP134 due to the presence of the respective deaminases (Table S1). Serine would be directly converted into pyruvate and ammonia by the action of serine deaminase whose gene is also found in the genomes of the rest of *Cupriavidus*/*Ralstonia* strains. On the other hand, threonine would be deaminated to 2-oxobutanoate by threonine deaminase that also seems to be encoded in the genomes of the rest of *Cupriavidus*/*Ralstonia* strains.

A complete bifurcated pathway for degradation of histidine is found in the genome of strain JMP134 consistent with its ability to grow using this amino acid as only carbon and energy source. Histidine catabolism proceeds in four or five steps pathways overlapping in the first three reactions to transform this amino acid into N-formimino-Lglutamate [43]. At this point, N-formimino-Lglutamate can be converted to L-glutamate via single- or two-step reactions. Both routes are encoded in the genome of *C. necator* JMP134 (Table S1) and in the genomes of the rest of *Cupriavidus* strains, but only the single-reaction route is encoded in the genomes of strains 12J and GMI1000.

The catabolism of branched-chain amino acids (BCAAs) starts by the action of an α-oxoglutarate-dependent aminotransferase which catalyzes the hydrolysis of leucine, isoleucine and valine to α-oxoisocaproate, α-oxo-γ-methylvalerate, and α-oxoisovalerate, respectively, followed by decarboxylation of these α-oxoacids to their corresponding branched chain acyl-CoA, in a reaction catalyzed by a branched chain α-oxoacid dehydrogenase complex. Both, the BCAA aminotransferase and the α-oxoacid dehydrogenase complex seem to be encoded in the genome of strain JMP134 (Table S1).

The catabolism of branched-chain amino acids (BCAA) starts with leucine dehydrogenase or α-oxoglutarate-dependent aminotransferase which catalyzes the hydrolysis isoleucine and valine to to the corresponding α-oxoacids (α-oxoisocaproate, α-oxo-γ-methylvalerate and α-oxoisovalerate, respectively). Subsequently, the branched-chain α-oxoacid dehydrogenase complex catalyzes the decarboxylation to the corresponding acyl-coenzyme A (CoA) derivatives [44]. Both BCAA aminotransferase and leucine dehydrogenase seems to be encoded in the genome of strain JMP134, in addition to the common branched-chain α-oxoacid dehydrogenase complex (Table S1). The branched-chain aa aminotransferase seems to be also encoded in the rest of *Cupriavidus* strains, but only strain H16 additionally encodes leucine dehydrogenase.

Finally, L-cysteine would be degraded by two alternative pathways in *C. necator* JMP134 since a L-cysteine desulfhydrase transforming L-cysteine to ammonia, hydrogen sulphide and pyruvate, and a Fe^2+^-dependent cysteine dioxygenase that performs sulfoxidation to form cysteine sulfinic acid, are found in the genome of this strain. Both enzymes seem to be conserved in the genomes of the rest of *Cupriavidus/Ralstonia* strains.

The pathways for the degradation of aromatic amino acids –tryptophan, phenylalanine and tyrosine– have been analyzed in detail, recently [19].

### Degradation of carbohydrates

*C. necator* JMP134 is very limited in sugar or sugar acids degradation, since only fructose and gluconate can be metabolized by this strain, in contrast with other *Cupriavidus* strains that are able to use glucose, 2-ketogluconate and N-acetyl-glucosamine [37]. Fructose and gluconate can be initially catabolized by fructokinase and gluconate kinase, respectively, using a Entner-Doudoroff pathway, with 2-keto-3-desoxy-6-phosphogluconate (KDPG) aldolase as key enzyme. The genes encoding this pathway are equally distributed in both chromosomes and several examples of gene redundancy are found (glucose-6-phosphate isomerase, glucose-6-phosphate 1-dehydrogenase, 6-phosphogluconolactonase and phosphogluconate dehydratase) (Table S1). It should be noted that similar genes encoding gluconate kinase are found in the rest of *Cupriavidus*/*Ralstonia* strains, but a homolog to fructokinase gene is only found in the genome of strain H16. In addition, genes encoding a glucosaminate deaminase and 2-keto-3-deoxygluconate kinase are found in the genome of strain JMP134 and in the rest of *Cupriavidus*/*Ralstonia* strains, putatively enabling the utilization of glucosaminate by these strains. However, the utilization of this sugar by strain JMP134 has not been evaluated [37].

Although glucose would be metabolized by strain JMP134, since a glucokinase gene is found in its genome, the absence of an uptake system for this hexose would explain why this strain does not use this sugar as a carbon source. In addition, the absence of 2-ketogluconate kinase and N-acetylglucosamine-6-phosphate deacetylase encoding genes is consistent with the inability of strain JMP134 to use these sugars as growth substrates. *C. necator* JMP134 has incomplete Embden-Meyerhoff-Parnas and oxidative pentose phosphate pathways due to the absence of genes encoding the key enzymes phosphofructokinase and 6-phosphogluconate dehydrogenase, respectively.

### Metabolism of polyhydroxyalkanoate (PHA)

The microbial polyesters as poly-(R)-3-hydroxybutyrate (PHB), belonging to the family of polyhydroxyalkanoic acids (PHA), occurred as insoluble inclusions in the cytoplasm and served as a storage compound for carbon and energy when the cells are cultivated under imbalanced growth conditions. The metabolism of PHA has been extensively studied in *C. necator* H16, a model for microbial polyoxoester production [45]. Analysis of genome sequence revealed that strain JMP134 possesses the key enzymes in PHA biosynthesis (Table S1): a type I poly(3-hydroxybutyrate) polymerase (Reut_A1347), two β-ketoacyl-CoA thiolases (Reut_A1348; Reut_A1353) and four NADPH-dependent β-ketoacyl-CoA reductases (Reut_A1349, Reut_B3865, Reut_C6018, Reut_B4127) which, together, convert acetyl-CoA into PHB. In addition to type I PHA synthase, strain JMP134 contains also a type II PHA synthase (Reut_A2138). Type II PHA synthases utilize thioesters of at least five carbon atoms whereas type I enzymes utilize thioesters of three to five carbon atoms. It should be noted that *C. necator* H16 lacks apparent type II PHA synthases. Additionally, four phasin (PHA-granule associated protein) encoding genes are found in the genome of strain JMP134. Phasins are most probably involved providing, together with phospholipids, a layer at the surface of the PHA granules [45]. Finally, the intracellular depolymerization of PHB in *C. necator* H16 is performed by multiple PHB depolymerases and PHB oligomer hydrolases [45]. Similarly, the mobilization of PHB in strain JMP134 seems to be performed by two putative PHB oligomer hydrolases (Reut_A1981, Reut_A1272) and five PHB depolymerases (Reut_A1049, Reut_A0762, Reut_B4702, Reut_B3626, Reut_B5113). Genes similar to the ones involved in PHB metabolism are found in all the rest of *Cupriavidus*/*Ralstonia* strains, indicating that this trait is widespread in these genera. It should be noted that PHB accumulation in *C. necator* JMP134 has been verified previously [46].

### Nitrogen metabolism

Among the genes participating in nitrogen metabolism found on chromosome 1 of *C. necator* JMP134 are Reut_A3432, a putative ammonium monooxygenase (*amoA*), and an NAD glutamate dehydrogenase (NAD-gdh; 1371497–1376338 bp) putatively involved in ammonification. The NAD-gdh protein has 55% and 57% amino acid identity with the NAD-gdh protein reported in *Azoarcus* sp. and *Pseudomonas aeruginosa*, respectively [47].

Denitrification is encoded by three gene clusters on chromosome 2. The nitrate reduction *nap* genes (Reut_B4761-4765) have >80% amino acid identity with the corresponding genes in *C. eutropha* H16 [48]; likewise, the nitrite reduction genes (Reut_B5010-5018) have >75% amino acid identity [49]; the *nor* genes catalyzing later steps in denitrification (Reut_B5055-5057) have >80% amino acid identity [50], [51]. Two nitrogen metabolism regulators, *narX* and *narL* (1804512–1807189 bp), also have high identity to their counterparts in *C. eutropha* H16.

### Aerobic energy metabolism

Genome analysis of strain JMP134 revealed a robust energy metabolism typical of most free-living heterotrophs dwelling in an environment with fluctuating O_2_ levels. The presence of an extensive inventory of genes for respiratory chain components including at least nine distinct terminal oxidases indicates that the aerobic respiration chain adapts to varying concentrations of O_2_. Genes required for formation of complexes I, II and III of oxidative phosphorylation are present in large chromosome of strain JMP134: (i) a typical proton-pumping NADH:quinone oxidoreductase encoded by a large cluster of 14 genes (Reut_A0961– Reut_A0974); (ii) a succinate dehydrogenase belonging to the four-subunit type C subgroup [52] encoded by four genes (Reut_A2322–Reut_A2325); and (iii) the cytochrome bc1 complex, coupling electron transfer from ubiquinol to periplasmic cytochromes c with proton pumping, encoded by three genes (Reut_A3091– Reut_A3093). All of these genes are highly conserved and share similarities to the relatives of *Cupriavidus*/*Ralstonia* group.

In addition to use of proton-translocating NADH dehydrogenase of complex I in energy production, strain JMP134 may employ two different type II NADH dehydrogenases (Reut_A0874/Reut_B4838) to optimize the (NADH)/(NAD+) balance under changing environmental conditions [53]. It should be noted that the second of these genes seems to be unique to strain JMP134, in contrast with the first one that is highly conserved in the rest of *Cupriavidus*/*Ralstonia* strains.

The respiratory chain of strain JMP134 can be fueled, besides NADH dehydrogenases, by at least three formate dehydrogenases allowing the use of formate as an auxiliary energy source by this strain [54], but not as a growth substrate since the product of formate oxidation, CO_2_ is not fixed by strain JMP134 [37]. A soluble, NAD^+^-reducing, molybdenum-containing formate dehydrogenase, previously characterized in strain *C. necator* H16 [55], is encoded by the five genes of the *fds* cluster located in large chromosome and seems to be conserved in all *Cupriavidus* strains, but not in *Ralstonia* genus (Table S1). Another soluble formate dehydrogenase may be encoded by *fdw* genes on small chromosome. The FdwA and FdwB gene products would form a dimeric tungsten-containing formate dehydrogenase that recycles NADH at the expense of formate oxidation to CO_2_, as proposed for *C. necator* H16 [56]. This soluble formate dehydrogenase is also found in *C. taiwanensis* LMG19424 (Table S1). An additional membrane-bound formate dehydrogenase is putatively encoded by *fdhA*, *fdhB* and *fdhC* genes, which would encode a catalytic subunit, an iron-sulfur subunit, and a transmembrane cytochrome b subunit, respectively, as proposed for *C. necator* H16 [56]. In addition, an accessory gene *fdhD* is found in this cluster located in large chromosome (Table S1). This kind of formate dehydrogenase seems to be encoded in the genomes of all the rest of *Cupriavidus*/*Ralstonia* strains. The presence of a second membrane-bound formate dehydrogenase encoded by *fdo* genes, as described in strain H16 [56], is not found in strain JMP134.

Strain JMP134 apparently contains an unusually large number of genes for terminal oxidases catalyzing the reduction of O_2_ to water using cytochrome c or quinol as electron donors: (i) one operon coding for an aa3-type cytochrome oxidase, which typically operates at high oxygen concentrations; (ii) one operon coding for a cbb3-type cytochrome oxidase having high affinity for oxygen, and qualifying to operates at extremely low pressures of oxygen; (iii) one operon for a bb3-type cytochrome oxidase; (iv) two operons coding for bd-type quinol oxidases; and (v) three operons coding for bo3-type quinol oxidases (Table S1). All these terminal oxidases-encoding operons are also found in strain H16 and its putative function has been analyzed, according to previous physiological and biochemical studies [56]. All the rest of *Cupriavidus*/*Ralstonia* strains have the aa3-, cbb3- and bb3-type cytochrome oxidases-encoding operons but a lower number of quinol oxidases-encoding operons (Table S1). Finally, it should be mentioned the presence of a putative caa3-type high-potential iron sulfur protein (HiPIP) oxidase-encoding operon, exclusively found in the genome of strain JMP134. The HiPIP is a small soluble protein functioning as the electron carrier between the cytochrome bc complex and the HiPIP terminal oxidase of the respiratory chain described in the strict aerobe and thermohalophile *Rhodothermus marinus* [57]. However, no homologous gene encoding a HiPIP similar to that described in *R. marinus* is found in the genome of strain JMP134, revealing that the identity of the putative electron donor for this terminal oxidase remains unknown in this bacterium.

Altogether, the genomic analysis of energy metabolism in strain JMP134 confirms that this bacterium is well adapted to life in habitats subject to fluctuating carbon sources and physicochemical conditions. The existence of putative ecoparalogs or isoenzymes having different kinetic properties (e.g., terminal oxidases) or metal cofactor content (e.g., formate dehydrogenases) allows this bacterium to cope with rapidly changing O_2_ concentrations and environments with varying metal supply.

### Quorum sensing

Although several quorum-sensing systems employing N-acyl-homoserine lactones (AHLs) have been identified in members of the closely related *Burkholderia* and *Ralstonia* genera [58], [59], none were detected in the *C. necator* JMP134 genome. On the other hand, a complete phenotype conversion (Phc) regulatory system was found to be encoded by chromosome 1. This system has been studied primarily in the phytopathogen *R. solanacearum* GMI1000 where it forms the core of the complex network that regulates virulence and pathogenicity genes [60]. At the center of this Phc system is PhcA, a LysR-type transcriptional regulator, and the products of the *phcBSRQ* operon that control levels of active PhcA in response to cell density. The unique signaling molecule employed for quorum sensing is the volatile 3-hydroxy palmitic acid methyl ester (3-OH PAME) [60]. 3-OH PAME post-transcriptionally modulates the activity of PhcA by acting as the signal for an atypical two-component regulatory system. This system consists of a membrane-bound sensor-kinase, PhcS, which phosphorylates PhcR, an unusual response regulator with a C-terminal kinase domain in place of a DNA-binding domain [60]. The amino acid identity between the *C. necator* JMP134 and the *R. solanacearum* GMI1000 Phc gene products range from 56% to 75%. The presence of a *phcA* ortholog in a *Cupriavidus* strain capable of fully complementing *R. solanacearum phcA* mutants was previously reported [61]. That strain also appears to make a form of 3-OH PAME and to contain orthologs of *phcB* and *phcS* [61]. The possible physiological functions regulated by the Phc system in *C. necator* JMP134 pose intriguing questions that are, as yet, unanswered.

### Plant-bacteria associations

Members of the genus *Cupriavidus*, as well as the closely related *Ralstonia* and *Burkholderia*, include a few plant pathogens and symbionts. There is substantial evidence suggesting that members of these two genera are able to interact with plants and to establish diverse commensal or even mutualistic associations with these hosts [62], [63], [64]. Although this area has not been the focus of research in *C. necator* JMP134, specifically, recent experimental evidence suggests that this bacterium is able to proliferate in the rhizosphere and even within internal tissues of *A. thaliana* (Zúñiga, A, Ledger, Th. and B. González, unpublished data). For most of the plant bacteria associations described so far, the bacterial genes typically involved include those encoding protein or nucleotide transport from the microorganism to the host, as well as those involved in the production of extracellular enzymes and the elicitors of the plant hypersensitive response [65], [66]. *C. necator* JMP134 has several genes related to protein transport. On chromosome 1 are found several genes related to type IV transport systems (Reut_A0401-0404, Reut_A0784-0788, Reut_A0779, Reut_A1436, Reut_A2960-2962, and Reut_A3131-3135). Reut_A2970 encodes a protein translocase with 72% amino acid identity to the SecA of *Burkholderia multivorans* ATCC 17616. Chromosome 2 also harbors a number of genes encoding putative components of a type IV secretion system (Reut_B5405-5416).

### Phage sequences

On chromosome 1 of *C. necator* JMP134 is found a large phage-like gene cluster that spans ∼43 kb and includes 55 CDSs (Reut_A2365-2419). Most of these putative proteins have no homologs in other sequenced genomes of members of the *Ralstonia* or the *Cupriavidus* genera. However, homologs for many of these proteins, with amino acid sequence identities >60%, are present in various *Burkholderia* species, including *B. vietnamiensis* G4, *B. cenocepacia* HI2424, *B. dolosa* AUO158, and *B. multivorans* ATCC 17616. The overall sequence identity and arrangement of the CDSs clustered in this region suggest that this putative phage is related to the characterized temperate *Burkholderia* podophage, BcepC6B.

A few additional phage-like sequences are found scattered in chromosomes 1 and 2. These include phage-type integrases (Reut_A0577, Reut_A1625, Reut_A2191, and Reut_B5345), two DNA polymerases with similarity to the DNA polymerase of phage SPO1 (Reut_A1937 and Reut_B4396), and two hypothetical phage proteins (Reut_A0552 and Reut_A2198). Since these sequences are not accompanied by other phage-like genes and are instead adjacent to transposon-related sequences, they likely correspond to transposon fragments rather than phage remnants. One possible exception: Reut_A2191 is accompanied by genes encoding putative phage regulatory proteins (Reut_A2193 and Reut_A2195) and thus might be descended from a prophage.

The megaplasmid contains a higher density of phage-type integrase genes and transposon elements than that found on either chromosome. There are five integrase sequences (Reut_C5954, Reut_C5993, Reut_C6147, Reut_C6164 and Reut_C6343) all of which are adjacent to transposons, thus suggesting that these integrases are part of transposon elements. This conclusion is further supported by the identification of one such sequence in plasmid pJP4 next to the transposase of a Tn3 family transposon (IS1071).

### Protein transport, adherence, motility

*C. necator* JMP134 has a complete *sec* general protein secretion system, including homologs of *secA* (Reut_A2970), *secY* (Reut_A3159), *secE* (Reut_A3195), *secG* (Reut_A0960), *secD* (Reut_A2810), *secF* (Reut_A2811) and *yajC* (Reut_A2809), as well as a signal peptidase (Reut_A2254). It also has all the components of the *sec*-independent twin-arginine translocation (TAT) system for protein translocation: *tatC* (Reut_A3098), *tatA*/*E* (Reut_A3100), *tatB* (Reut_3099), and *tatD*-related components (Reut_A1437 and Reut_A1078). The TAT system is distinguished by the ability to translocate fully-folded proteins and is found also in *C. eutropha* H16, *C. metallidurans* CH34, and *R. solanacearum* GMI1000. Complete type II and type IV secretion systems are also present in these four organisms. In contrast, of the four, only the plant pathogen *R. solanacearum* GMI1000 possesses a type III secretion system.

A full set of *che* genes encoding chemotaxis functions forms a putative operon on chromosome 2 adjacent to *fla* genes encoding the flagellum and motor proteins. Additional copies of all except two of the *che* genes (*cheY* and *cheZ*) are scattered on chromosome 1. These genes are also located on chromosome 2 in *C. eutropha* H16 and *C. metallidurans* CH34.

### Conclusions

Analysis of the complete genome of *C. necator* JMP134 adds further insights into the evolution of multipartite genomes in β-proteobacteria, and the presence of aromatic catabolism and other metabolic functions. It has been proposed that multipartite genomes arise through intragenomic gene transfer between progenitor chromosomes and ancestral plasmids. Our analysis supports that hypothesis and further indicates that distinct plasmids served as the scaffolds for the assembly of secondary chromosomes in the *Cupriavidus*, *Ralstonia*, and *Burkholderia* lineages. Furthermore, both chromosomes in the *Cupriavidus* show evidence of significant gene duplication and lateral gene transfer, with foreign DNA preferentially incorporated into the secondary chromosomes. The *C. necator* JMP134 genome contains nearly 300 genes potentially involved in the catabolism of aromatic compounds and encodes almost all of the central ring-cleavage pathways. Although all these genomes possess a significant number of aromatic catabolism functions, including central and peripheral pathways, the genome of strain JMP134 is by far the one that provides more versatile degradative abilities. The availability of the complete genome sequence for *C. necator* JMP134 provides the groundwork for further elucidation of the mechanisms and regulation of chloroaromatic compound biodegradation, and its interplays with several other key metabolic processes analyzed here.

## Supporting Information

Table S1 Functional annotation of key metabolic genes of C. necator JMP134.
(0.27 MB DOC)

Figure S1 Functional distribution of unique genes. COG categories are as follows: Information storage and processing: A, RNA processing, modification; B, chromatin structure; J, translation, ribosomal structure/biogenesis; K, transcription; L, DNA replication, recombination, repair. Cellular processes: D, cell division, chromosome partitioning; M, cell envelope biogenesis outer membrane; N, Cell motility and secretion; P, Inorganic ion transport and metabolism; T, Signal transduction mechanisms. Metabolism: C, Energy production and conversion; G, Carbohydrate transport and metabolism; E, Amino acid transport and metabolism; F, Nucleotide transport and metabolism; H, Coenzyme metabolism; I, Lipid metabolism; Q, Secondary metabolites biosynthesis, transport and catabolism; Poorly characterized: R, General function prediction only; S, Function unknown.
(2.77 MB TIF)
