# Supplementary material for: The Complete Multipartite Genome Sequence of Cupriavidus necator JMP134, a Versatile Pollutant Degrader
Source: PLoS One. 2010 Mar 22;5(3):e9729. doi: 10.1371/journal.pone.0009729 (PMC2842291; doi:10.1371/journal.pone.0009729)
Supplement: Table S1 — Functional annotation of key metabolic genes of C. necator JMP134. (0.27 MB DOC) [file pone.0009729.s001.doc]

**Table S1.** Functional annotation of key metabolic genes of *C. necator* JMP134

| **Additional metabolic capabilities** | |  |  |  | | |  |
| --- | --- | --- | --- | --- | --- | --- | --- |
|  |  |  |  | Related gene product | | | |
| Locus Tag | Function | Identity | Gene | Accession | | Microorganism | |
| Reut_B3918 | Cyclohexanecarboxylate-CoA ligase | 382/545 (70%) | *aliA* | NP_946004 | | *R. palustris* CGA009 | |
| Reut_B3917 | Cyclohexanecarboxyl-CoA dehydrogenase | 316/383 (82%) | *aliB* | NP_946005 | | *R. palustris* CGA009 | |
| Reut_B3916 | 2-ketocyclohexanecarboxyl-CoA hydrolase | 210/260 (80%) | *badI* | NP_946006 | | *R. palustris* CGA009 | |
| Reut_B3915 | 2-hydroxycyclohexanecarboxyl-CoA dehydrogenase | 196/255 (76%) | *badH* | NP_946007 | | *R. palustris* CGA009 | |
| Reut_A1461 | Tetrahydrofurfuryl alcohol dehydrogenase | 597/708 (84%) | *tfaA* | AAF86335 | | *C. necator* Bo | |
| Reut_B4160 | Quinohaemoprotein ethanol dehydrogenase | 497/664 (74%) | *qhedh* | Q46444 | | *C. testosteroni* ATCC 15667 | |
| Reut_C6172 | Acetone carboxylase, beta subunit | 497/708 (70%) | *acxA* | YP_001418395 | | *X. autotrophicus* Py2 | |
| Reut_C6173 | Acetone carboxylase, alpha subunit | 532/761 (69%) | *acxB* | YP_001418396 | | *X. autotrophicus* Py2 | |
| Reut_C6174 | Acetone carboxylase, gamma subunit | 100/165 (60%) | *acxC* | YP_001418397 | | *X. autotrophicus* Py2 | |
|  |  |  |  |  | |  | |
| **Amino acids degradation** | |  |  |  | |  | |
|  |  |  |  | Related gene product | | | |
| Locus Tag | Function | Identity | Gene | Accession | | Microorganism | |
| Reut_A1812 | L-Asparaginase, type II | 204/343 (59%) | ansA | NP_744601 | | P. putida KT2440 | |
| Reut_B3724 | Glutamate dehydrogenase | 303/443 (68%) | gdhA | NP_742836 | | P. putida KT2440 | |
| Reut_A2735 | L-Aspartate oxidase | 346/527 (65%) | nadB | NP_743584 | | P. putida KT2440 | |
| Reut_A1791 | Aspartate ammonia-lyase | 287/470 (61%) | aspA | NP_747439 | | P. putida KT2440 | |
| Reut_A3340 | Bifunctional proline dehydrogenase/pyrroline-5-carboxylate dehydrogenase | 977/1325 (73%) | putA | NP_747050 | | P. putida KT2440 | |
| Reut_B5637 | Histidine ammonia-lyase | 405/509 (79%) | hutH | YP_002870047 | | P. fluorescens SBW25 | |
| Reut_A2716 | Histidine ammonia-lyase | 287/490 (58%) | hutH | YP_002870047 | | P. fluorescens SBW25 | |
| Reut_A0896 | Urocanate hydratase | 498/556 (89%) | hutU | YP_002870041 | | P. fluorescens SBW25 | |
| Reut_B5636 | Urocanate hydratase | 498/555 (89%) | hutU | YP_002870041 | | P. fluorescens SBW25 | |
| Reut_A2715 | Urocanate hydratase | 411/549 (74%) | hutU | YP_002870041 | | P. fluorescens SBW25 | |
| Reut_A2713 | Imidazolone-5-propionate hydrolase | 246/395 (62%) | hutI | YP_002870049 | | P. fluorescens SBW25 | |
| Reut_A2712 | N-formimino-L-glutamate deiminase | 243/463 (52%) | hutF | YP_002870038 | | P. fluorescens SBW25 | |
| Reut_A2711 | N-formylglutamate amidohydrolase | 133/257 (51%) | hutG | YP_002870050 | | P. fluorescens SBW25 | |
| Reut_A1591 | Formimidoylglutamase | 153/302 (50%) | hutG' | NP_251865 | | P. aeruginosa PAO1 | |
| Reut_B5816 | Leucine dehydrogenase | 164/327 (50%) | bcd | NP_390288 | | B. subtilis 168 | |
| Reut_C6219 | Leucine dehydrogenase | 157/345 (45%) | bcd | NP_390288 | | B. subtilis 168 | |
| Reut_B4282 | Branched-chain amino acid aminotransferase | 153/340 (45%) | ybgE | NP_388121 | | B. subtilis 168 | |
| Reut_B4976 | Branched-chain alpha-keto acid dehydrogenase E1 component alpha chain | 116/338 (34%) | bkdA1 | AAA65614 | | P. putida PpG2 | |
| Reut_B4977 | Branched-chain alpha-keto acid dehydrogenase E1 component beta chain | 147/336 (43%) | bkdA2 | AAA65616 | | P. putida PpG2 | |
| Reut_B4978 | Branched-chain alpha-keto acid dehydrogenase E2 component | 139/418 (33%) | bkdB | AAA65617 | | P. putida PpG2 | |
| Reut_A1466 | Isovaleryl-CoA dehydrogenase | 285/388 (73%) | liuA/ivdA | NP_250705 | | P. aeruginosa PAO1 | |
| Reut_A0135 | Isovaleryl-CoA dehydrogenase | 285/388 (73%) | liuA/ivdA | NP_250705 | | P. aeruginosa PAO1 | |
| Reut_B4257 | Branched-chain acyl-CoA dehydrogenase | 232/371 (62%) | scdA/acdH | NP_251242 | | P. aeruginosa PAO1 | |
| Reut_A3329 | Glycine cleavage system T protein (Aminomethyltransferase) | 196/380 (51%) | gcvT | CAA52144 | | Escherichia coli W3110 | |
| Reut_A3330 | Glycine cleavage system H protein | 70/122 (57%) | gcvH | CAA52145 | | Escherichia coli W3110 | |
| Reut_A3331 | Glycine dehydrogenase (decarboxylating) | 566/963 (58%) | gcvP | CAA52146 | | Escherichia coli W3110 | |
| Reut_A3333 | L-serine deaminase I | 247/457 (54%) | sdaA | AP_002433 | | Escherichia coli W3110 | |
| Reut_B4831 | L-serine deaminase II | 244/463 (52%) | sdaB | AP_003363 | | Escherichia coli W3110 | |
| Reut_A0413 | Threonine deaminase | 259/516 (50%) | ilvA | AP_004024 | | Escherichia coli W3110 | |
| Reut_B4909 | Threonine deaminase | 262/512 (51%) | ilvA | AP_004024 | | Escherichia coli W3110 | |
| Reut_B5878 | L-Alanine dehydrogenase | 255/371 (68%) | ald | AAC23578 | | Shewanella sp. Ac10 | |
| Reut_A1168 | L-Alanine dehydrogenase | 229/370 (61%) | ald | AAC23578 | | Shewanella sp. Ac10 | |
| Reut_A1934 | Alanine racemase | 209/357 (58%) | dadX | AP_001815 | | Escherichia coli W3110 | |
| Reut_A2530 | Alanine racemase | 195/363 (53%) | dadX | AP_001815 | | Escherichia coli W3110 | |
| Reut_A2547 | D-Alanine dehydrogenase | 282/419 (67%) | dadA | AP_001814 | | Escherichia coli W3110 | |
| Reut_B5045 | Cysteine dioxygenase | 101/179 (56%) | cdo | ACB72254 | | Variovorax paradoxus TBEA6 | |
| Reut_A1058 | L-Cysteine desulfhydrase | 280/402 (69%) | iscS | AAT78348 | | Pseudomonas putida TS1138 | |
| Reut_C6331 | L-Cysteine desulfhydrase | 276/401 (68%) | iscS | AAT78348 | | Pseudomonas putida TS1138 | |
|  |  |  |  |  | |  | |
| **Carbohydrates degradation** | |  |  |  | |  | |
|  |  |  |  | Related gene product | | | |
| Locus Tag | Function | Identity | Gene | Accession | | Microorganism | |
| Reut_A1648 | Fructokinase | 247/302 (81%) | frcK | YP_841020 | | *C. necator* H16 | |
| Reut_A1649 | Glucose-6-phosphate isomerase | 452/535 (84%) | pgi2 | YP_841019 | | *C. necator* H16 | |
| Reut_A1386 | Glucose-6-phosphate isomerase | 458/541 (84%) | pgi1 | YP_726002 | | *C. necator* H16 | |
| Reut_A1650 | Glucose-6-phosphate 1-dehydrogenase | 429/482 (89%) | zwf2 | YP_841018 | | *C. necator* H16 | |
| Reut_B5329 | Glucose-6-phosphate 1-dehydrogenase | 450/485 (92%) | zwf3 | YP_842078 | | *C. necator* H16 | |
| Reut_B5327 | Glucokinase | 263/333 (78%) | glk | YP_842076 | | *C. necator* H16 | |
| Reut_B5328 | 6-Phosphogluconolactonase | 175/225 (77%) | pgl | YP_842077 | | *C. necator* H16 | |
| Reut_B4086 | 6-Phosphogluconolactonase | 108/231 (46%) | pgl | YP_842077 | | *C. necator* H16 | |
| Reut_B5330 | Phosphogluconate dehydratase | 554/611 (90%) | edd2 | YP_842079 | | *C. necator* H16 | |
| Reut_A1081 | Phosphogluconate dehydratase | 557/612 (91%) | edd1 | YP_725687 | | *C. necator* H16 | |
| Reut_B4024 | 2-Keto-3-deoxy-6-phosphogluconate aldolase | 186/212 (87%) | eda | YP_840733 | | *C. necator* H16 | |
| Reut_A1082 | Gluconate kinase | 138/168 (82%) | gntK | YP_725688 | | *C. necator* H16 | |
| Reut_B4022 | Deaminase | 378/422 (89%) | H16_B1211 | YP_840731 | | *C. necator* H16 | |
| Reut_B4023 | 2-Keto-3-deoxygluconate kinase | 287/311 (92%) | kdgK | YP_840732 | | *C. necator* H16 | |
|  |  |  |  |  | |  | |
| **PHA metabolism** | |  |  |  | |  | |
|  |  |  |  | Related gene product | | | |
| Locus Tag | Function | Identity | Gene | Accession | Microorganism | | |
| Reut_A1347 | Type I poly(3-hydroxybutyrate) polymerase | 498/594 (83%) | *phaC1* | YP_725940 | *C. necator* H16 | | |
| Reut_A2138 | Type II poly(3-hydroxybutyrate) polymerase | 282/541 (52%) | *phaC1* | AAK19605 | *P. pseudoalcaligenes* HBQ06 | | |
| Reut_A1348 | β-Ketoacyl-CoA thiolase | 373/392 (95%) | *phaA* | YP_725941 | *C. necator* H16 | | |
| Reut_A1353 | β-Ketoacyl-CoA thiolase | 372/394 (94%) | *bktB* | YP_725948 | *C. necator* H16 | | |
| Reut_A1349 | β-Ketoacyl-CoA reductase | 237/246 (96%) | *phaB1* | YP_725942 | *C. necator* H16 | | |
| Reut_B3865 | β-Ketoacyl-CoA reductase | 225/236 (95%) | *phaB3* | YP_726636 | *C. necator* H16 | | |
| Reut_C6018 | β-Ketoacyl-CoA reductase | 177/236 (75%) | *phaB3* | YP_726636 | *C. necator* H16 | | |
| Reut_B4127 | β-Ketoacyl-CoA reductase | 172/236 (72%) | *phaB3* | YP_726636 | *C. necator* H16 | | |
| Reut_B3864 | Phasin (PHA-granule associated protein) | 115/158 (72%) | *phaP3* | YP_726637 | *C. necator* H16 | | |
| Reut_C6019 | Phasin (PHA-granule associated protein) | 115/175 (65%) | *phaP2* | NP_942840 | *C. necator* H16 | | |
| Reut_B4349 | Phasin (PHA-granule associated protein) | 96/159 (60%) | *phaP3* | YP_726637 | *C. necator* H16 | | |
| Reut_B4922 | Phasin (PHA-granule associated protein) | 62/151 (41%) | *phaP2* | NP_942840 | *C. necator* H16 | | |
| Reut_A1049 | Intracellular polyhydroxyalkanoate depolymerase | 385/412 (93%) | *phaZ1* | YP_725659 | *C. necator* H16 | | |
| Reut_A0762 | Intracellular polyhydroxyalkanoate depolymerase | 326/404 (80%) | *phaZ2* | YP_727307 | *C. necator* H16 | | |
| Reut_B4702 | Intracellular polyhydroxyalkanoate depolymerase | 164/377 (43%) | *phaZ3* | YP_728504 | *C. necator* H16 | | |
| Reut_B3626 | Polyhydroxyalkanoate depolymerase | 274/365 (75%) | *phaZ6* | YP_841585 | *C. necator* H16 | | |
| Reut_B5113 | Polyhydroxyalkanoate depolymerase | 243/361 (67%) | *phaZ7* | YP_841913 | *C. necator* H16 | | |
| Reut_A1981 | D-(-)-3-hydroxybutyrate oligomer hydrolase | 518/674 (76%) | *phaY1* | YP_726716 | *C. necator* H16 | | |
| Reut_A1272 | D-(-)-3-hydroxybutyrate oligomer hydrolase | 250/293 (85%) | *phaY2* | YP_725843 | *C. necator* H16 | | |
|  |  |  |  |  |  | | |
| **Energy metabolism** | |  |  |  |  | | |
|  |  |  |  | Related gene product | | | |
| Locus Tag | Function | Identity | Gene | Accession | Microorganism | | |
| Reut_A0616 | Transcriptional regulator of formate dehydrogenase operon | 320/360 (88%) | fdsR | YP_725155 | *C. necator* H16 | | |
| Reut_A0617 | NAD-dependent formate dehydrogenase gamma subunit | 146/179 (81%) | fdsG | YP_725156 | *C. necator* H16 | | |
| Reut_A0618 | NAD-dependent formate dehydrogenase beta subunit | 478/517 (92%) | fdsB | YP_725157 | *C. necator* H16 | | |
| Reut_A0619 | NAD-dependent formate dehydrogenase alpha subunit | 884/957 (92%) | fdsA | YP_725158 | *C. necator* H16 | | |
| Reut_A0620 | Formate dehydrogenase accessory protein | 247/287 (86%) | fdsC | YP_725159 | *C. necator* H16 | | |
| Reut_A0621 | NAD-dependent formate dehydrogenase delta subunit | 64/74 (86%) | fdsD | YP_725160 | *C. necator* H16 | | |
| Reut_A0682 | Formate dehydrogenase alpha subunit | 941/1012 (92%) | fdhA1 | YP_727381 | *C. necator* H16 | | |
| Reut_A0683 | Formate dehydrogenase iron-sulfur subunit | 193/195 (98%) | fdhB1 | YP_727380 | *C. necator* H16 | | |
| Reut_A0685 | Cytochrome b subunit of formate dehydrogenase | 319/402 (79%) | fdhC | YP_727378 | *C. necator* H16 | | |
| Reut_A0687 | Protein required for formate dehydrogenase activity | 253/276 (91%) | fdhD1 | YP_727376 | *C. necator* H16 | | |
| Reut_B4651 | Tungsten-containing formate dehydrogenase alpha subunit | 861/937 (91%) | fdwA | YP_841215 | *C. necator* H16 | | |
| Reut_B4652 | Tungsten-containing formate dehydrogenase beta subunit | 490/569 (86%) | fdwB | YP_841216 | *C. necator* H16 | | |
| Reut_A0314 | aa3-type cytochrome oxidase, subunit II | 378/421 (89%) | ctaC | YP_724861 | *C. necator* H16 | | |
| Reut_A0315 | aa3-type cytochrome oxidase, subunit I | 519/535 (97%) | ctaD | YP_724862 | *C. necator* H16 | | |
| Reut_A0316 | cytochrome C oxidase assembly protein | 173/203 (85%) | ctaG | YP_724864 | *C. necator* H16 | | |
| Reut_A0318 | aa3-type cytochrome oxidase, subunit III | 272/286 (95%) | ctaE | YP_724866 | *C. necator* H16 | | |
| Reut_A2042 | cbb3-type cytochrome oxidase maturation protein | 46/48 (95%) | *ccoS* | YP_726784 | *C. necator* H16 | | |
| Reut_A2041 | cbb3-type cytochrome c oxidase subunit I | 471/482 (97%) | *ccoN* | YP_726783 | *C. necator* H16 | | |
| Reut_A2040 | cbb3-type cytochrome oxidase, monoheme subunit II | 203/220 (92%) | *ccoO* | YP_726782 | *C. necator* H16 | | |
| Reut_A2039 | cbb3-type cytochrome oxidase, subunit III | 46/54 (85%) | *ccoQ* | YP_726781 | *C. necator* H16 | | |
| Reut_A2038 | cbb3-type cytochrome oxidase, diheme subunit IV | 257/305 (84%) | *ccoP* | YP_726780 | *C. necator* H16 | | |
| Reut_A2037 | Polyferredoxin | 432/490 (88%) | *ccoG* | YP_726779 | *C. necator* H16 | | |
| Reut_B3632 | bb3-type cytochrome oxidase, subunit II | 321/439 (73%) | *coxM* | YP_841574 | *C. necator* H16 | | |
| Reut_B3633 | bb3-type cytochrome oxidase, subunit I | 550/574 (95%) | *coxN* | YP_841573 | *C. necator* H16 | | |
| Reut_B3634 | bb3-type cytochrome oxidase, subunit III | 172/215 (80%) | *coxO* | YP_841572 | *C. necator* H16 | | |
| Reut_B3635 | bb3-type cytochrome oxidase, subunit III | 192/212 (90%) | *coxP* | YP_841571 | *C. necator* H16 | | |
| Reut_B3636 | bb3-type cytochrome oxidase, subunit IV | 102/126 (80%) | *coxQ* | YP_841570 | *C. necator* H16 | | |
| Reut_A0982 | bo3-type quinol oxidase, subunit II | 273/323 (84%) | *cyoA1* | YP_725580 | *C. necator* H16 | | |
| Reut_A0983 | bo3-type quinol oxidase, subunit I | 628/657 (95%) | *cyoB1* | YP_725581 | *C. necator* H16 | | |
| Reut_A0984 | bo3-type quinol oxidase, subunit III | 180/213 (84%) | *cyoC1* | YP_725582 | *C. necator* H16 | | |
| Reut_A0985 | bo3-type quinol oxidase, subunit IV | 84/120 (70%) | *cyoD1* | YP_725583 | *C. necator* H16 | | |
| Reut_B4579 | bo3-type quinol oxidase, subunit II | 202/283 (71%) | *cyoA2* | YP_726143 | *C. necator* H16 | | |
| Reut_B4578 | bo3-type quinol oxidase, subunit I | 568/657 (86%) | *cyoB2* | YP_726142 | *C. necator* H16 | | |
| Reut_B4577 | bo3-type quinol oxidase, subunit III | 131/182 (71%) | cyoC2 | YP_726141 | *C. necator* H16 | | |
| Reut_B4576 | bo3-type quinol oxidase, subunit IV | 86/131 (65%) | cyoD2 | YP_726140 | *C. necator* H16 | | |
| Reut_B4890 | bo3-type chinol oxidase, subunit II | 219/303 (72%) | *cyoA3* | YP_729181 | *C. necator* H16 | | |
| Reut_B4891 | bo3-type chinol oxidase, subunit I | 576/667 (86%) | *cyoB3* | YP_729182 | *C. necator* H16 | | |
| Reut_B4892 | bo3-type chinol oxidase, subunit III | 167/229 (72%) | *cyoC3* | YP_729183 | *C. necator* H16 | | |
| Reut_B4893 | bo3-type chinol oxidase, subunit IV | 96/131 (73%) | *cyoD3* | YP_729184 | *C. necator* H16 | | |
| Reut_B4284 | bd-type quinol oxidase subunit I | 421/480 (87%) | *cydA1* | YP_840698 | *C. necator* H16 | | |
| Reut_B4285 | bd-type quinol oxidase subunit II | 303/335 (90%) | *cydB1* | YP_840697 | *C. necator* H16 | | |
| Reut_B4101 | bd-type quinol oxidase subunit I | 423/483 (87%) | *cydA2* | YP_840979 | *C. necator* H16 | | |
| Reut_B4100 | bd-type quinol oxidase subunit II | 304/335 (90%) | *cydB2* | YP_840980 | *C. necator* H16 | | |
| Reut_B4435 | HiPIP oxidase, subunit II | 120/300 (40%) | *rcoxA* | CAC08531 | R. marinus PRQ- 62B | | |
| Reut_B4436 | HiPIP oxidase, subunit I | 321/536 (59%) | *rcoxB* | CAC08532 | R. marinus PRQ- 62B | | |
| Reut_B4437 | HiPIP oxidase, subunit III | 82/207 (39%) | *rcoxC* | CAC08533 | R. marinus PRQ- 62B | | |
| Reut_B4438 | HiPIP oxidase, subunit IV | 24/59 (40%) | *rcoxD* | CAC08534 | R. marinus PRQ- 62B | | |
