# Supplementary figures and images for: The Complete Multipartite Genome Sequence of Cupriavidus necator JMP134, a Versatile Pollutant Degrader
Source: PLoS One. 2010 Mar 22;5(3):e9729. doi: 10.1371/journal.pone.0009729 (PMC2842291; doi:10.1371/journal.pone.0009729)

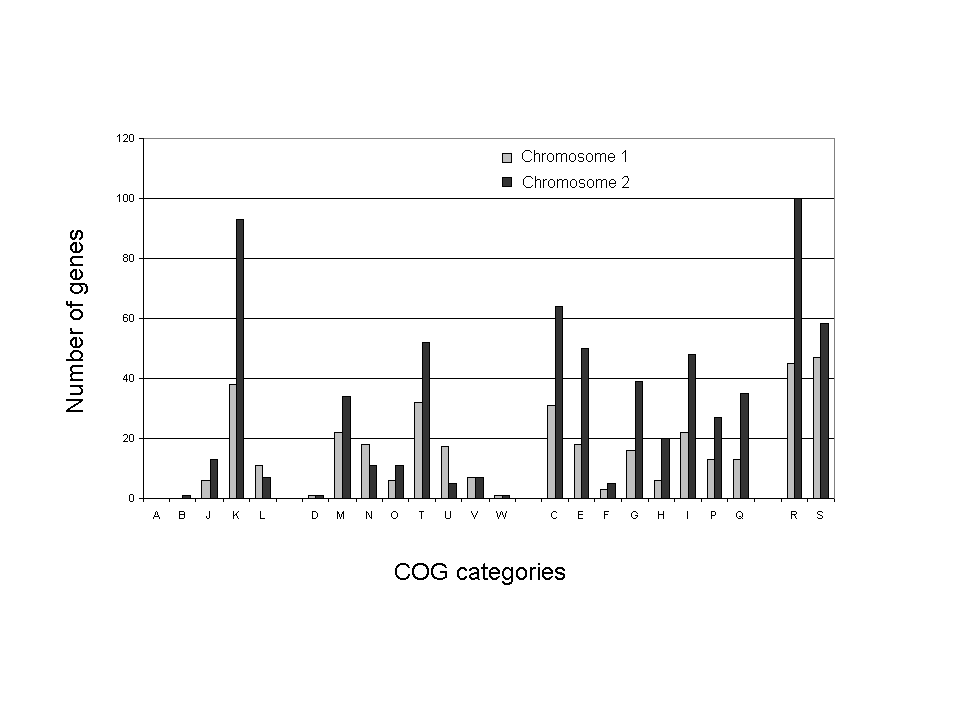

Supplement: Figure S1 — Functional distribution of unique genes. COG categories are as follows: Information storage and processing: A, RNA processing, modification; B, chromatin structure; J, translation, ribosomal structure/biogenesis; K, transcription; L, DNA replication, recombination, repair. Cellular processes: D, cell division, chromosome partitioning; M, cell envelope biogenesis outer membrane; N, Cell motility and secretion; P, Inorganic ion transport and metabolism; T, Signal transduction mechanisms. Metabolism: C, Energy production and conversion; G, Carbohydrate transport and metabolism; E, Amino acid transport and metabolism; F, Nucleotide transport and metabolism; H, Coenzyme metabolism; I, Lipid metabolism; Q, Secondary metabolites biosynthesis, transport and catabolism; Poorly characterized: R, General function prediction only; S, Function unknown. (2.77 MB TIF) [file pone.0009729.s002.tif]
